# Lichen-Derived Actinomycetota: Novel Taxa and Bioactive Metabolites

**DOI:** 10.3390/ijms24087341

**Published:** 2023-04-16

**Authors:** Qingrong Yang, Zhiqiang Song, Xinpeng Li, Yage Hou, Tangchang Xu, Shaohua Wu

**Affiliations:** Yunnan Institute of Microbiology, School of Life Sciences, Yunnan University, Kunming 650091, China

**Keywords:** actinomycetes, antimicrobial, bioactive secondary metabolites, biosynthetic pathways, diversity, lichen

## Abstract

Actinomycetes are essential sources of numerous bioactive secondary metabolites with diverse chemical and bioactive properties. Lichen ecosystems have piqued the interest of the research community due to their distinct characteristics. Lichen is a symbiont of fungi and algae or cyanobacteria. This review focuses on the novel taxa and diverse bioactive secondary metabolites identified between 1995 and 2022 from cultivable actinomycetota associated with lichens. A total of 25 novel actinomycetota species were reported following studies of lichens. The chemical structures and biological activities of 114 compounds derived from the lichen-associated actinomycetota are also summarized. These secondary metabolites were classified into aromatic amides and amines, diketopiperazines, furanones, indole, isoflavonoids, linear esters and macrolides, peptides, phenolic derivatives, pyridine derivatives, pyrrole derivatives, quinones, and sterols. Their biological activities included anti-inflammatory, antimicrobial, anticancer, cytotoxic, and enzyme-inhibitory actions. In addition, the biosynthetic pathways of several potent bioactive compounds are summarized. Thus, lichen actinomycetes demonstrate exceptional abilities in the discovery of new drug candidates.

## 1. Introduction

Lichens form important symbiotic communities in the ecosystem and are characterized by a symbiotic association between fungi and algae. They occupy 8% of the earth’s surface [1]. Some bioactive compounds, such as usnic acid, gyrophoric acid, diffractaic acid, polysaccharides, anthraquinones, and terpenes, have been isolated from lichens, and some of these compounds have been employed in clinical treatments [2]. Organisms with a slower growth rate reportedly exhibit strong resistance to external secondary metabolism [3]. Further, organisms that move slowly and thrive in low-resource environments produce large amounts of defensive metabolites for protection against their many predators. Lichens and their symbiotic organisms, especially actinomycetes, grow slowly. They are natural habitats for the production of beneficial bioactive compounds or metabolites.

Antibiotics produced by microorganisms contribute significantly to human health. Actinomycetes are an essential resource for the discovery of drug-lead compounds. Actinomycete drug resources have been utilized and developed for many years. Hence, identifying new active structural substances has become increasingly difficult [4]. Lichens are a unique group of organisms formed by symbioses between fungi and algae or cyanobacteria [5]. The wide variety of lichens can provide new sources of actinomycetes for use in the discovery of novel drugs [6]. Only a few national and international research groups have investigated actinomycete lichen resources. Moreover, few groups have reported active metabolites derived from actinomycetes. 

Lichen-derived actinomycetota are potent producers of bioactive metabolites. This review summarizes the compounds isolated from lichen-derived actinomycetes. The compounds are classified into 11 types based on their different structures. Some secondary metabolites exhibit various biological activities, such as anti-inflammatory, antimicrobial, anticancer, cytotoxic, and enzyme-inhibitory activities. The chemical structures of 114 secondary metabolites isolated from lichen-associated actinomycetes and 25 novel actinomycetota species are listed in this review. Furthermore, the biological activities of some lichen actinomycetota have also been investigated, although the effective chemical components of these strains are still unknown. The biosynthetic pathways of some unique secondary metabolites from lichen-derived actinomycetes are also reported here.

## 2. Novel Actinomycetota Taxa

**Figure 1:**
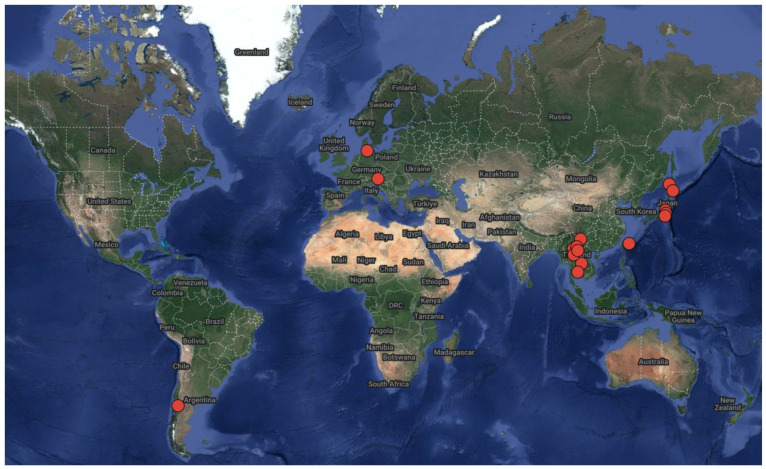
Collection points for lichen samples (the red dots represent the sampling sites).

**Figure 2:**
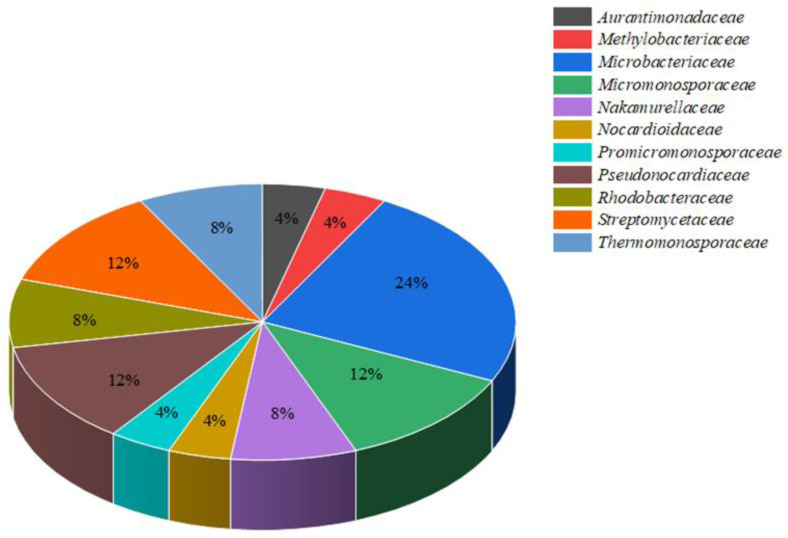
The proportions of novel actinomycete species belonging to different families.

The phylum “Actinobacteria” was modified to “Actinomycetota” by Goodfellow in 2021 [7]. Therefore, we use “actinomycetota” in the present article. Actinomycetes are extensively dispersed. The 25 novel species isolated from lichens between 2007 and 2022 are described in Table 1. In Figure 1, the red dots indicate the collection sites of the lichen samples from which new species of actinomycetes were identified. Most published literature suggests the distribution of new lichen-associated actinomycetota in Asia, especially in Yunnan Province, China. Among the 25 isolated species, 6 actinomycete species (24%) belonged to the family *Microbacteriaceae*. Three (12%) species belonged to each of the families of *Micromonosporaceae*, *Pseudonocardiaceae*, and *Streptomycetaceae*. The remaining actinomycetes came from the families *Nakamurellaceae*, *Rhodobacteraceae*, *Streptomycetaceae*, and others (Figure 2).

The humic acid–vitamin agar selective medium was mainly used to isolate most of the novel actinomycetota species (Table 1). A few novel species were isolated using standard growth media such as ISP2 medium and potato dextrose agar. Most species were incubated for 1–4 weeks at 25–30 °C.

## 3. Natural Products from Lichen-Associated Actinomycetota

A total of 114 compounds from lichen-associated actinomycetes were reported. Based on their structural characteristics, they were classified into diketopiperazines, peptides, indoles, furanones, quinones, isoflavonoids, etc. Some exhibit anti-inflammatory, antimicrobial, anticancer, cytotoxic, and enzyme-inhibitory activities. These compounds are promising small molecules for advancement as new drug and pesticide candidates.

### 3.1. N-Containing Compounds

#### 3.1.1. Diketopiperazines

Diketopiperazines are the smallest cyclic dipeptides formed by the double condensation of two *α*-amino acids. They have a stable six-membered ring that serves as an important pharmacophore [32]. Diketopiperazines are structures with numerous biological functions of interest to natural product researchers.

**Figure 3:**
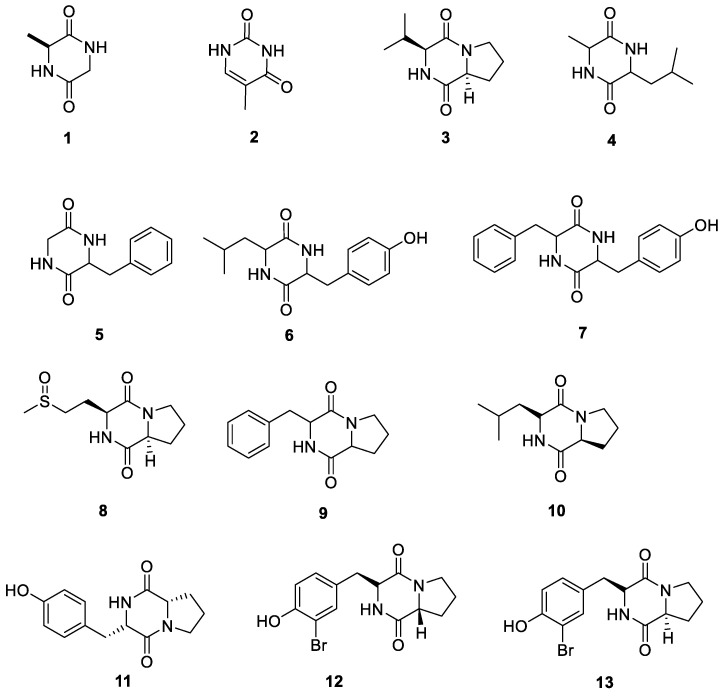
Chemical structures of compounds **1**–**13** from lichen-associated actinomycetota.

Cyclo (Gly-L-Ala) (**1**) and 5-methyl-uracil (**2**) were isolated from the actinomycete *Amycolatopsis* sp. YIM 130687, collected from the Jinsha River region in Yunnan Province [33]. Compound **1** exhibited weak activity against *Escherichia coli* and *Salmonella typhimurium*, and **2** showed weak inhibitory activity against *S. typhimurium*. Cyclo (L-Pro-L-Val) (**3**) was isolated from *Nocardia ignorata* [34]. Cyclo-(Ala-Leu) (**4**), cyclo-(Gly-Phe) (**5**), cyclo-(Leu-Tyr) (**6**), and cyclo-(Phe-Tyr) (**7**) were obtained from *Amycolatopsis* sp. YIM 130932, associated with the lichen *Punctelia rudecta* found in Yunnan Province [35]. Cyclo (L-Pro-L-OMet) (**8**) was isolated from *Nocardia ignorata* [34]. Cyclo-(Phe-Pro) (**9**) and cyclo-(L-Leu-L-Pro) (**10**) were isolated from *Streptomyces cyaneofuscatus* MOLA 1488, associated with the marine lichen *Lichina confinis* [36]. The crude extract of this strain showed anticancer activity against murine melanoma cells (B16 cell line) and the HaCaT cell line (normal immortalized keratinocyte cell line). The half maximal inhibitory concentration (IC_50_) values for B16 and HaCaT cell lines were 0.33 ± 0.2 μM and 0.25 ± 0.1 μM, respectively. Cyclo-(Pro-Tyr) (**11**) was isolated from the actinobacterium QHHL-09 from the Tibetan Plateau [37]. Two new brominated diketopiperazines, cyclo (D-Pro-L-Br-Tyr) (**12**) and cyclo (L-Pro-L-Br-Tyr) (**13**), were isolated from *Nocardia ignorata* [34]. All 13 diketopiperazines from the lichen derived actinomycetota described above are presented in Figure 3.

#### 3.1.2. Peptides

Peptide natural products (PNPs) represent a unique class of compounds with fascinating structural motifs that impart important biological activities [38]. Cis-3-isobutyl-tetrahydroimidazo [1,2-a] pyridine-2,5-dione (**14**) was isolated from the lichen-associated actinobacterium QHHL-09, found on the Tibetan Plateau. It inhibited HIV-1 reverse transcriptase, glutamate receptor 2, and protein kinase ck2 [37]. Turnagainolide B (**15**) was obtained from the strain *Streptomyces* sp. YIM 130597 from the lichen *Punctelia rudecta*, found in Yunnan Province, China [35]. It displayed weak antibacterial activity toward *S. typhimurium* with a MIC value of 64 μg/mL.

**Figure 4:**
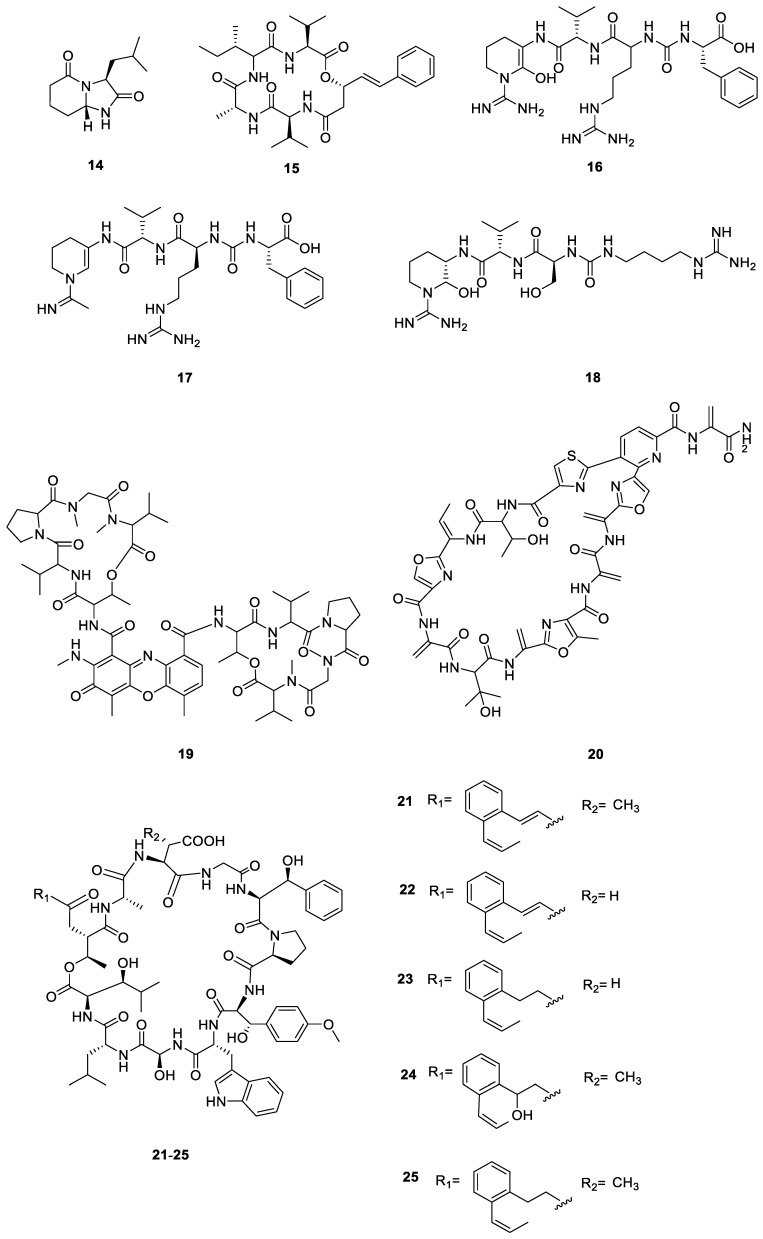
Chemical structures of compounds **14**–**25** from lichen-associated actinomycetota.

Antipain (**16**), V2 (antipain dehydration product) (**17**), and lichostatinal (**18**) were isolated from a British Columbian lichen associated with *Streptomycetes* sp. L91-3 [39]. Compound **16** was a known potent cathepsin K (CatK) inhibitor, whereas **17** was a new dehydrated analog of antipain and a much weaker CatK inhibitor. Compound **18** was identified as a new potent CatK inhibitor using affinity crystallography. *N*-methyldactinomycin (**19**) was collected from *Streptomyces cyaneofuscatus* MOLA1488 from Erquy (France) [36]. Geninthiocin B (**20**) was isolated from *Streptomyces* sp. YIM 130001 obtained from the tropical rainforest in Xishuangbanna (Yunnan, China). It showed antibacterial activity against *Bacillus subtilis* [28]. Skyllamycins A–E (**21**–**25**) were isolated from a *Streptomyces anulatus* strain from the New Zealand lichen *Pseudocyphellaria dissimilis*. Antibacterial assays revealed that compound **24** possessed superior activity against *B. subtilis* E168 compared to previously reported congeners [40]. All 12 PNPs from the lichen derived actinomycetota described above are presented in Figure 4.

#### 3.1.3. Indoles

**Figure 5:**
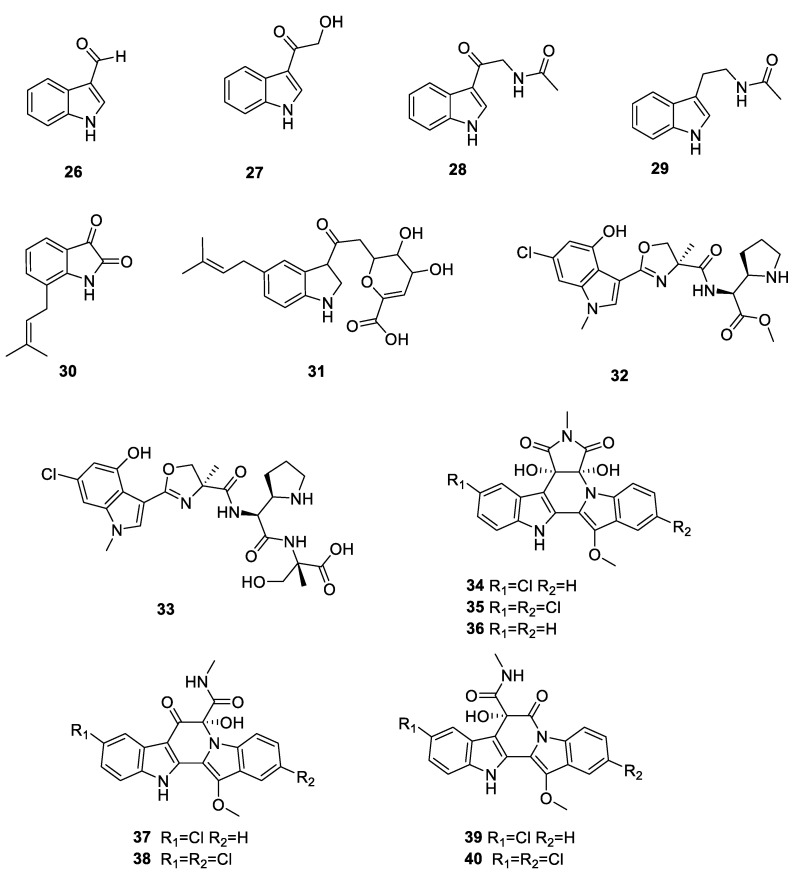
Chemical structures of compounds **26**–**40** from lichen-associated actinomycetota.

Due to its diverse biological activities, the indole scaffold is a vital heterocyclic organic compound of medical and pharmaceutical interest [41]. Indole-carboxaldehyde (**26**) was isolated from the lichen *Collema auriforme*, found in the Austrian town of Kesselfallklamm [34]. Compound **26** showed weak cytotoxic activity against the HaCaT (IC_50_ = 79 ± 6 μM) and B16 (IC_50_ = 72 ± 6 μM) cell lines.

(3-Hydroxyacetyl) indole (**27**) and *N*-acetyl-β-oxotryptamine (**28**) were isolated from *Lichina confinis*, a marine lichen collected on the Brittany coast (Erquy, France) [36]. Acetotryptamide (**29**) was isolated from the fresh lichen *Punctelia borreri* from the Jinsha River region, Yunnan Province. It possessed antibacterial activity against *S. typhimurium* and *E. coli* [33]. 7-Prenylisatin (**30**), 5-isoprenylindole-3-carboxylate (**31**), JBIR-126 (**32**), and JBIR-149 (**33**) were isolated from culturable lichen actinomycetes found around Qinghai Lake on the Qinghai–Tibet Plateau [37]. Seven new compounds, cladoniamides A–G (**34**–**40**) were isolated from cultures of *Streptomyces uncialis* found on the surface of the lichen *Cladonia uncialis* collected near Pitt River, British Columbia. Cladoniamide G displayed significant cytotoxicity against human breast cancer MCF-7 cells in vitro at 10 μg/mL [42]. All 15 indoles from the lichen derived actinomycetota described above are presented in Figure 5.

#### 3.1.4. Pyrrole Derivatives

**Figure 6:**
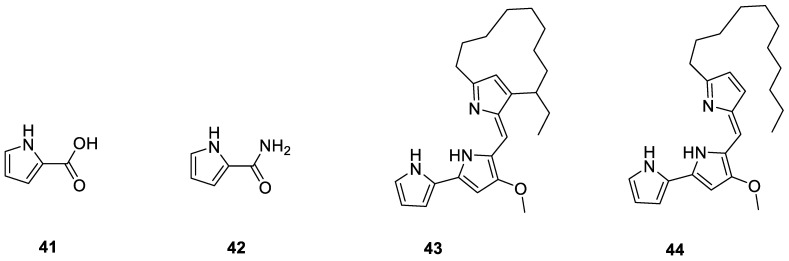
Chemical structures of compounds **41**–**44** from lichen-associated actinomycetota.

Pyrrole derivatives are a distinct class of heterocycle compounds that contribute significantly to natural products [43]. Mminaline (**41**) and 1H-pyrrole-2-carboxamide (**42**) were isolated from *Amycolatopsis* sp. YIM 130687, isolated from the fresh lichen *Punctelia borreri* found in the Jinsha River region, Yunnan Province. Compound **41** displayed weak antibacterial activity against MRSA, and **42** showed weak antibacterial activity against *Staphylococcus aureus* and *F. solani* [33]. Metacycloprodigiosin (**43**) and undecylprodigiosin (**44**) were extracted from the QHHL-18 isolate associated with lichens found around the Qinghai Lake on the Qinghai–Tibet Plateau [37]. All 4 pyrrole derivatives from the lichen derived actinomycetota described above are presented in Figure 6.

#### 3.1.5. Pyridine Derivatives

**Figure 7:**
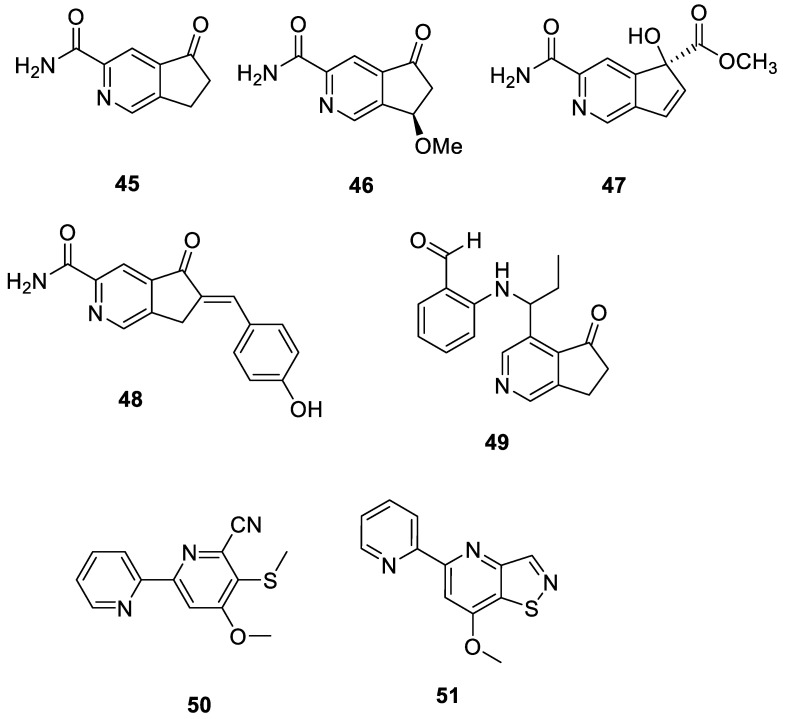
Chemical structures of compounds **45**–**51** from lichen-associated actinomycetota.

Pyridine is a crucial heterocyclic framework found in natural products. Methods have been developed for pyridine synthesis because of their importance and appeal in organic chemistry and natural product research [44]. Four new echinosporins, amycolasporins A–D (**45**–**48**), were derived from the lichen-associated actinomycete *Amycolatopsis hippodrome*. Compounds **46** and **47** demonstrated antibacterial activity against *B. subtilis*, *S. aureus*, and *E. coli* [45]. A novel compound, JBIR-120 (**49**), was isolated from *Streptomyces* sp. RI104-LiC104 from a lichen found on Rishiri Island, Hokkaido Prefecture, Japan [46]. It was weakly cytotoxic against 22Rv1 cells (human prostate carcinoma epithelial cell line) and effectively inhibited the growth of cells activated by dihydrotestosterone. Two known compounds, 4-methoxy-5-(methylthio)-[2,2′-bipyridine]-6-carbonitrile (**50**) and 7-methoxy-5-(pyridin-2-yl) isothiazolo [4,5-b] pyridine (**51**), were isolated from the *Streptomyces* strain YIM 130597 collected from the lichen *Punctelia rudecta* in Yunnan, China. Compound **51** exhibited strong antibacterial activity against *S. aureus* (MIC value of 64 μg/mL), *E. coli* (MIC value of 32 μg/mL), *S. typhimurium* (MIC value of 64 μg/mL), and MRSA (MIC value of 32 μg/mL) [35]. All 7 pyridine derivatives from the lichen derived actinomycetota described above are presented in Figure 7.

#### 3.1.6. Aromatic Amides and Amines

One or both primary amino groups and the imino group in an aliphatic polyamine can interact with different acids, resulting in mono-, di-, or tri-substituted amide derivatives [47]. A new compound, (E)-3- hydroxy-2,4-dimethylhept-4-enamide (**52**), was derived from the marine actinomycete *Streptomyces cavourensis* YY01-17 [48]. 2-Acetamidophenol (**53**), phenacetamide (**54**), anthranilic acid (**55**), 4-(3-methylbut-2-enyloxy) benzamide (**56**), and 2-pyruvoylaminobenzamide (**57**) were isolated from the fresh lichen *Punctelia borreri*. Compound **53** effectively inhibited the growth of MCF-7 breast cancer cells. Compound **54** showed inhibitory activity against *F. graminearum* with a MIC value of 2 μg/mL and against *S. aureus* with a MIC value of 8 μg/mL [33]. Compounds **58** 3-(4-hydroxyphenyl)-*N*-methylpropanamide and **59** *N*-(4-hydroxyphenethyl)-acetamide were isolated from actinomycetes from the Qinghai–Tibet Plateau near Qinghai Lake [37]. Amycophthalazinone A (**60**) was a new phthalazinone derivative isolated from *Amycolatopsis* sp. YIM 130642. It exhibited inhibitory activity against *S. aureus*, *S. typhi*, and *Candida albicans* with MIC values of 32, 32, and 64 μg/mL, respectively [49].

**Figure 8:**
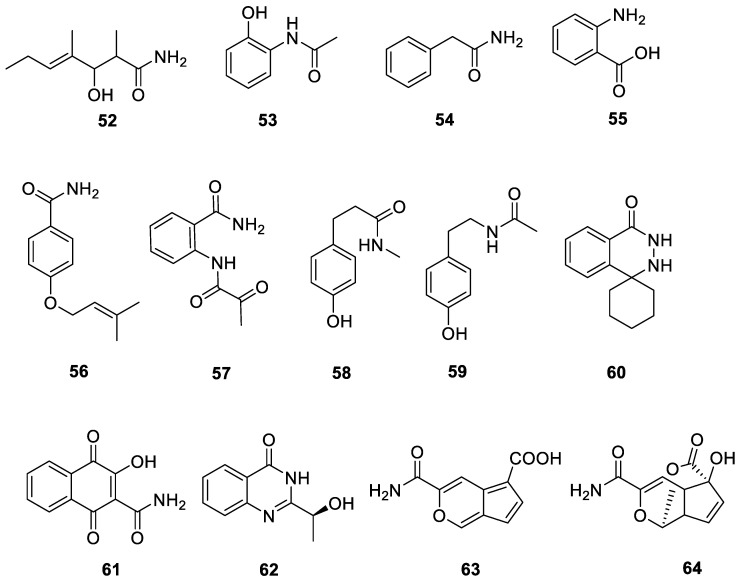
Chemical structures of compounds **52**–**64** from lichen-associated actinomycetota.

Compounds **61** 2-carbamoyl-3-hydroxy-1,4-naphthoquinone and **62** (–)-chry-sogine were isolated from the fresh lichen *Punctelia borreri* found in the Jinsha River region of Yunnan. Compound **61** displayed antimicrobial activity against *Botrytis cinerea*, *F. graminearum*, *S. aureus*, and MRSA with MIC values of 1, 1, 2, and 2 μg/mL, respectively [33]. A new echinosporin derivative, amycolasporin E (**63**), and a known echinosporin (**64**) were obtained from the lichen-associated actinomycete *Amycolatopsis* sp. YIM 130415 [45]. All 13 aromatic amides and amines from the lichen derived actinomycetota described above are presented in Figure 8.

### 3.2. Furanones

**Figure 9:**
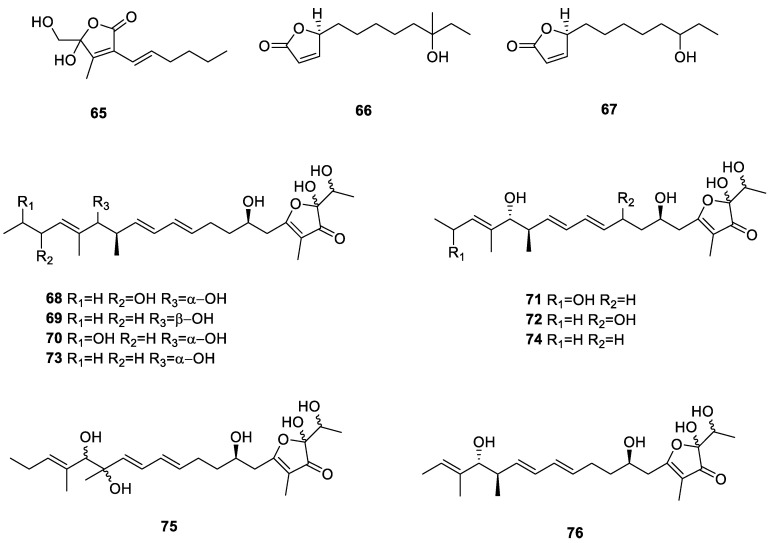
Chemical structures of compounds **65**–**76** from lichen-associated actinomycetota.

Furanones are commonly utilized in synthesis. The products display important pharmacological properties such as antiviral, anticancer, and antimicrobial properties [50]. JBIR-89 (**65**) was a new butenolide from the lichen-derived *Streptomyces* sp. RI104-LiB101 collected from Rishiri Island, Hokkaido Prefecture, Japan [51]. (5S)-5-(6-Hydroxy-6-methyloctyl)-furan-2(5H)-one (**66**) and (5S)-5-(6-hydroxy-7- methyloctyl)-furan-2(5H)-one (**67**) were produced by lichen-associated actinomycetes collected on the Tibetan Plateau, China [37]. Six new compounds, actinofuranones D-I (**68**–**73**), and three known compounds, JBIR-108 (**74**), E-975 (**75**), and E-492 (**76**), were obtained from *S. gramineus* derived from the lichen *Leptogium trichophorum* collected from an evergreen broad-leaf forest in Benzilan, Diqing, Yunnan, China [52]. Compounds **71**, **72**, **75**, and **76** inhibited nitric oxide synthase expression (in OS) in RAW 264.7 cells after LPS induction. In addition, **71**, **72**, **75**, and **76** inhibited the LPS-induced proinflammatory cytokines interleukin-6 (IL-6) and tumor necrosis factor *α* (TNF-*α*). All 12 furanones from the lichen derived actinomycetota described above are presented in Figure 9.

### 3.3. Aromatic Compounds

#### 3.3.1. Quinones

Quinones exhibit various biological activities, including antibacterial, antiplasmodial, antioxidant, trypanocidal, anticancer, and anti-HIV activities. All these activities are linked to the redox properties of their carbonyl groups [53]. Compound **77** (+)-4-hydroxy-1-teralone was obtained from the lichen-derived actinomycete *Amycolatopsis* sp. YIM 130687 collected from Yunnan Province, China [33]. Four novel nanomycin compounds, 4a*β*,10a*α*-dihydroxynanaomycin *β*A (**78**), 4a*β*,10a*β*-dihydroxynanaomycin *β*A (**79**), 4a*α*,10a*β*-dihy-droxynanaomycin *β*A (**80**), and 10*β*-hydroxynanaomycin *α*E (**81**), and two known compounds, nanaomycin *α*A (**82**) and nanaomycin *β*A (**83**), were produced by *Streptomyces hebeiensis* [54]. Compounds **82** and **83** displayed antibacterial activity against *S. aureus* and *B. subtilis* with MIC values ranging from 3.13 to 100 μg/mL, and modest antifungal activity against *C. albicans*.

Compound **84** 4-deoxy-*ε*-pyrromycinone was isolated from lichen actinomycetes collected around Qinghai Lake [37]. JBIR-88 (**85**) was a new angucycline produced by the lichen-derived *Streptomyces* spp. RI104-LiC106, and it exhibited antibacterial activity against *Micrococcus luteus*. Furthermore, **85** showed cytotoxicity against HeLa cells with a MIC of 36 μM and ACC-MESO-1 cells with a MIC of 52 μM [51]. BE-24566B (**86**) was a new antibiotic produced by *Streptomyces violaceusniger* A24566, which was isolated from a lichen collected in Jyogasaki, Shizuoka Prefecture, Japan. This compound inhibited *Gram*-positive bacteria, including methicillin-resistant *S. aureus* [55].

**Figure 10:**
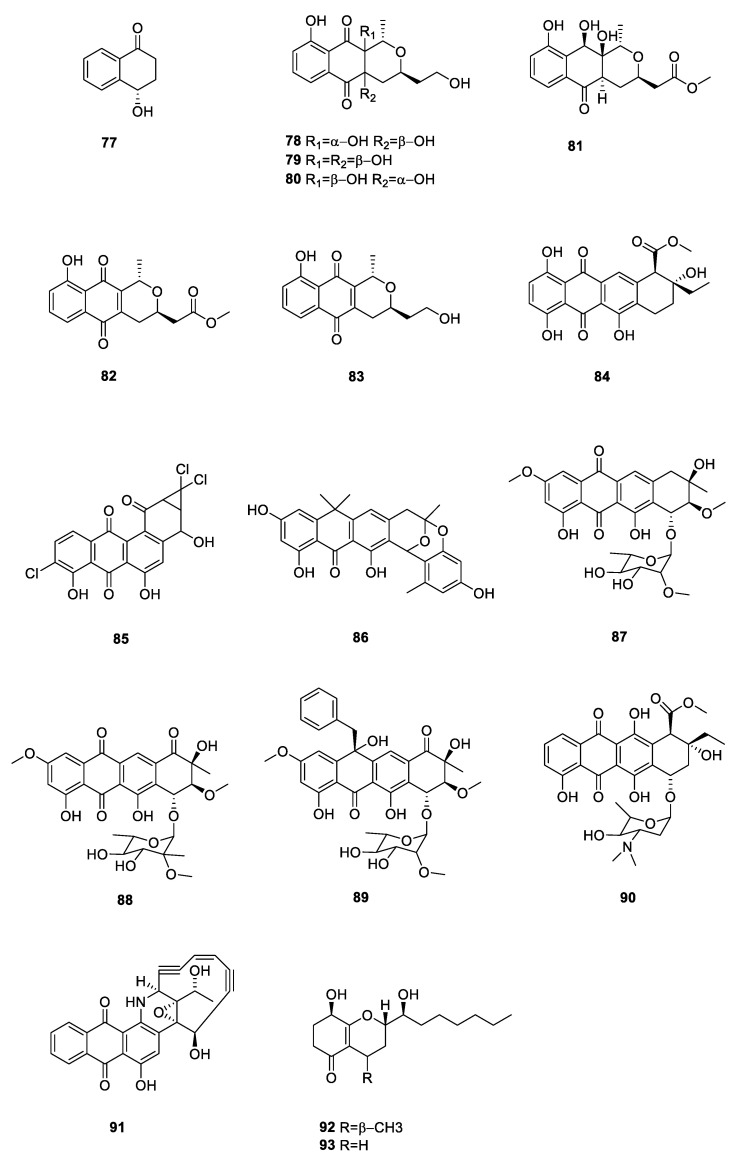
Chemical structures of compounds **77**–**93** from lichen-associated actinomycetota.

The lichen-derived actinomycete *Steptomyces* sp. 0630c, collected from Zhaosu County of the Xinjiang Uygur Autonomous Region, China, yielded three compounds, steffimycin D (**87**), steffimycin E (**88**), and steffimycin F (**89**) [56]. Compound **89** was a new steffimycin-type antibiotic with weak cytotoxicity towards MCF-7 (human breast adenocarcinoma), HepG-2 (human liver hepatocellular carcinoma), and A2780 (human ovarian carcinoma) cell lines. Two known compounds, **88** and **89**, exhibited potent antibacterial action against *S. aureus* with MIC values of 2 μg/mL. (7S*, 9R*, 10R*)-Pyrromycin (**90**) was isolated from a lichen actinomycete collected from Qinghai Lake [37]. Uncialamycin (**91**) was a novel enediyne antibiotic isolated from the British Columbian lichen *Cladonia uncialis* collected near the Pitt River [57]. It exhibited antibacterial activity against *S. aureus*, *E coli*, and *Burkholderia cepacia* with MIC values of 0.0000064, 0.002, and 0.001 μg/mL, respectively. 7-*O*-methylkoninginin D (**92**) and koninginin E (**93**) were obtained from *Streptomyces* sp. from the lichen *Punctelia rudecta* collected in Yunnan Province, China [35]. All 17 quinones from the lichen derived actinomycetota described above are presented in Figure 10.

#### 3.3.2. Isoflavonoids

**Figure 11:**
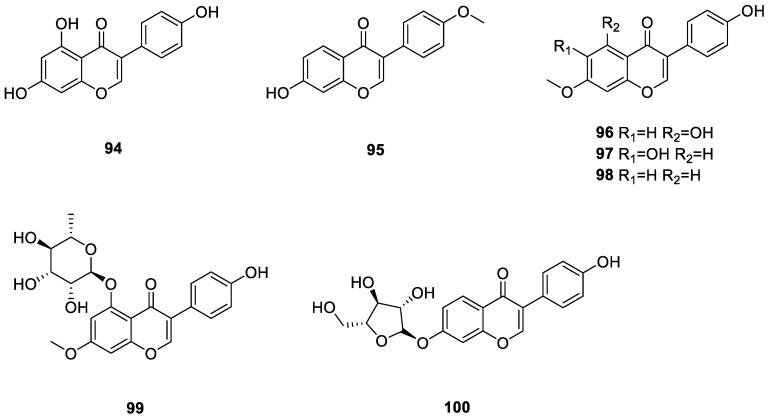
Chemical structures of compounds **94**–**100** from lichen-associated actinomycetota.

Isoflavonoids have a B-ring connected to their C-ring at the C-3 position (3-phenylchroman skeleton). They display a wide range of biological activities including antioxidant, anticarcinogenic, and antiproliferative properties. They also possess the ability to reduce osteoporosis and cardiovascular disease [58]. Seven isoflavonoid glycosides, namely genistein (**94**), formononetin (**95**), prunetin (**96**), kakkatin (**97**), isoformononetin (**98**), 7-*O*-methyl-5-*O*-α-L-rhamnopyranosylgenestein (**99**), and 7-*O*-α-D-arabinofur-anosyldaidzein (**100**), were isolated from *Amycolatopsis* sp. YIM 130642. Compounds **94** and **99** inhibited *S. aureus* and *E. coli.* Compound **96** inhibited *E. coli* with a MIC of 32 μg/mL, while **100** demonstrated bacteriostatic activity against *S. typhi* with a MIC value of 64 μg/mL [49]. All 7 isoflavonoids from the lichen derived actinomycetota described above are presented in Figure 11.

#### 3.3.3. Phenolic Derivatives

**Figure 12:**
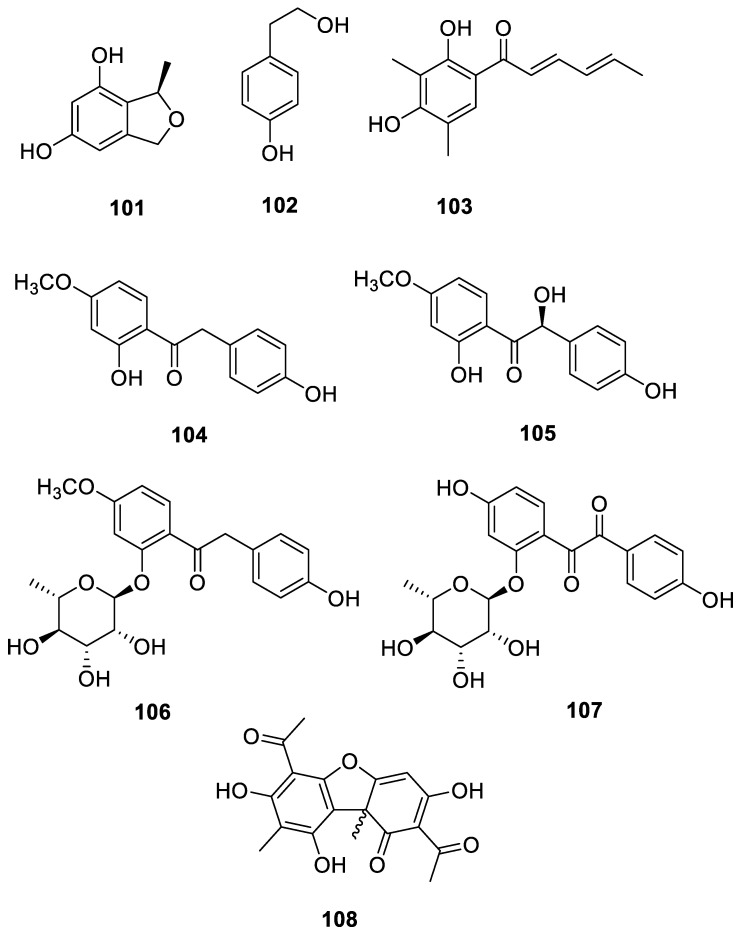
Chemical structures of compounds **101**–**108** from lichen-associated actinomycetota.

Phenolic compounds occur mainly in soluble conjugate and insoluble forms and are covalently bound to sugar moieties or structural components of the cell wall. Phenolic compounds have been extensively studied due to their varied health benefits as antioxidants and their roles in preventing chronic inflammation, cardiovascular disease, cancer, and diabetes [59,60]. The novel compound (*R*)-3-methyl-1,3-dihydroisobenzofuran-4,6-diol (**101**) was obtained from *Amycolatopsis hippodrome* [45]. *P*-hydroxyphenethyl alcohol (**102**) and sorbicillin (**103**) were obtained from *Amycolatopsis* sp. YIM 130687. Compound **103** showed cytotoxicity against the hepatocellular carcinoma cell line QGY-7703 and inhibited the growth of *C. albicans* [33]. 2-(4-Hydroxybenzylacetone)-5-methoxyphenol (**104**), amycolabenzoyl (**105**), and amycolabenzosides A–B (**106**–**107**) were obtained from *Amycolatopsis hippodrome*. Compound **104** attenuated nitric oxide production by suppressing the expression of nitric oxide synthase (iNOS) in LPS-induced RAW 264.7 cells in a dose-dependent manner [44]. Usnic acid (**108**), a prevalent cytotoxic secondary metabolite in lichens, was isolated from *Streptomyces cyaneofuscatus* MOLA1488. It imparts a green to greenish yellow color to many lichens [36]. All 8 phenolic derivatives from the lichen derived actinomycetota described above are presented in Figure 12.

### 3.4. Linear Esters and Macrolides

**Figure 13:**
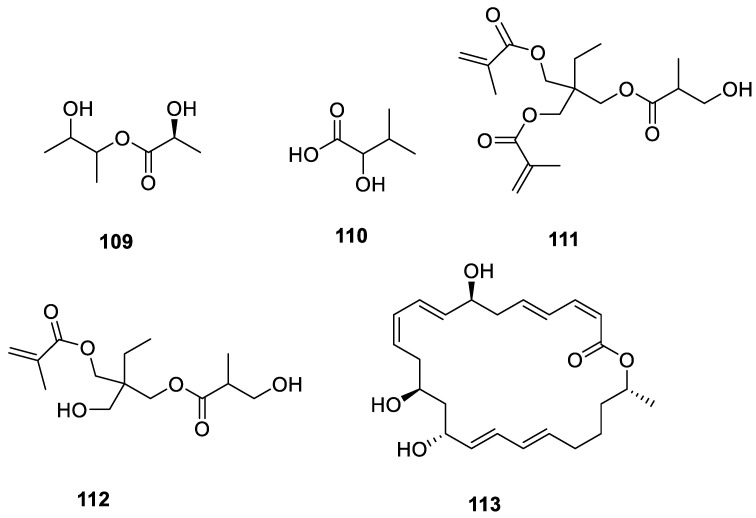
Chemical structures of compounds **109**–**113** from lichen-associated actinomycetota.

Esters of linear long-chain unsaturated fatty acids with multiple alcohols, both linear and branched, are widely used in the lubricant industry [61]. Macrolides are a large and structurally diverse class of macrocyclic natural products. They are valuable targets in synthetic chemistry due to their biological and medicinal importance [62]. One new linear compound, 2(S)-3′-hydroxybutan-2′-yl 2-hydroxypropanoate (**109**), and a known compound, 2-hydroxy-3-methylbutanoic acid (**110**), were procured from the marine-derived actinomycete *Streptomyces cavourensis* YY01-17 [48]. Cyaneodimycin (**111**) and cyaneomycin (**112**) were isolated from marine-lichen-associated *Streptomyces cyaneofuscatus*. Compound **111** exhibited antiproliferative action against B16, HaCaT, and Jurkat cell lines with MIC values of 27 ± 4 μM, 47 ± 11 μM, and 18.5 ± 0.5 μM, respectively [35]. Macrolactin A (**113**) was isolated from a lichen actinomycete found on the Qinghai–Tibet Plateau [37]. All 5 linear esters and macrolides from the lichen derived actinomycetota described above are presented in Figure 13.

### 3.5. Sterols

**Figure 14:**
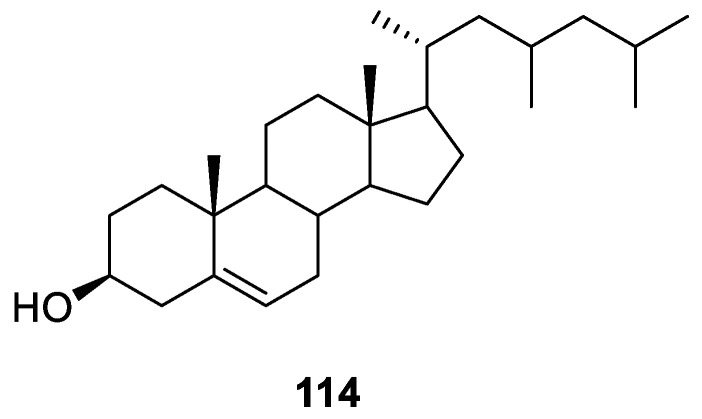
Chemical structure of compound **114** from lichen-associated actinomycetota.

Sterols are isoprenoid derivatives and structural components of biological membranes. They are currently being investigated for their structural, functional, and regulatory roles [63]. Campesterol (**114**) from the lichen-derived strain *Amycolatopsis* sp. YIM 130687 inhibited the growth of MRSA with a MIC of 128 μg/mL [33]. 1 sterol from the lichen derived actinomycetota described above are presented in Figure 14.

## 4. Bioactivity of Uncharacterized Compounds

The biological activities of many lichen actinomycetota have been investigated. However, these studies did not report any pure compounds or their structures. Several lichen-associated actinomycetota have been screened for their biological activities, such as antibacterial and antifungal activities, inhibition of β-glucosidase activity, etc. Such screenings without the structural elucidation of bioactive metabolites may not be useful for the discovery of new compounds. Nonetheless, these data highlight the possible utility of lichen-associated novel actinomycetota for the discovery of novel bioactive chemicals in the future [64].

Twelve actinomycete strains were isolated from lichens collected from the Maha Sarakham Province, Thailand. Among these, four *Streptomyces* isolates, LDG1-03, LDG1-15, LDG1-16, and LLG1-03, showed antimicrobial activity against *B. subtilis* ATCC 6633. LDG1-03 and LDG1-15 exhibited antimicrobial activity against *S. aureus* ATCC 25923, *Kocuria rhizophila* ATCC 9341, and *C. albicans* ATCC 10231. The *Actinoplanes* isolate LDG1-06 inhabited *C. albicans* ATCC 10231 [65]. *Actinomycetes* LC-23 was isolated from a lichen found growing on the bark of the *Averrhoa carambola* plant. Actinomycete pure strains were screened using agar diffusion on ISP2 agar medium to determine antimicrobial potency. The ethyl acetate extract of this strain displayed a positive inhibitory effect against *S. aureus* BTCC B-611 and *M. luteus* BTCC B-552 [66]. Lichen-associated *Streptomyces olivaceus* LEP7 was recovered from tree bark collected in the botanical garden of Nilgiris, Tamil Nadu, India. The extract of *Streptomyces* sp. LEP7 inhibited *E. coli*, *S. aureus*, and *P. aeruginosa* efficiently. The extract was found to contain cyclopentene upon GC-MS analysis. According to the report, the remarkable antimicrobial activity of *Streptomyces olivaceus* when tested against wound infections caused by microbial pathogens, and the derivation of cyclopentene from LEP7is a first step in this direction [67].

Extracts from the lichen *Umbilicaria esculenta* strongly inhibited mold and mammalian disaccharide hydrolytic enzymes (β-glucosidase). The inhibitory component of the extract was very stable, retaining more than 95% of its activity when treated with heat, acid, alkali, and some hydrolytic enzymes [68]. *Streptomyces* sp. DPUA 1542 and *Nocardia* sp. DPUA 1571, two actinomycetota strains isolated from Amazon River basin lichens, produced β-lactamase inhibitors which cured bovine mastitis [69]. *Streptomyces* sp. DPUA 1576, isolated from an Amazon basin lichen, yielded a fibrinolytic protease. This protease could potentially provide new and unexploited fibrinolytic enzymes for different therapeutic purposes [70,71].

## 5. Biosynthetic Pathways of Lichen Secondary Metabolites 

Advances in synthetic biology and associated technologies such as DNA synthesis, sequencing, and analysis techniques have accelerated the DBT cycle for metabolic and protein engineering to the point where both can be deployed to engineer the biosynthesis of a particular molecule [72]. The genes encoding these natural products in actinomycetes tend to be clustered, which allows the transfer of entire biosynthetic pathways to an exogenous host for heterologous expression. This strategy also enables the genetic modifications of such pathways, allowing the generation of various natural product analogs as well as the optimization of production yield [73].

**Figure 15:**
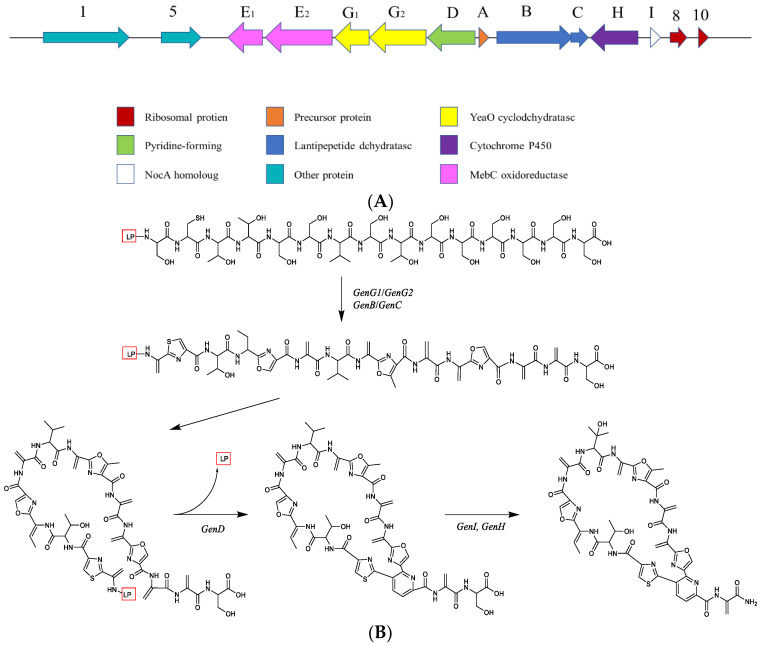
(**A**) The gene clusters of *Streptomyces* sp. YIM130001. (**B**) The biosynthetic pathway of geninthiocin B [28].

The production of the novel thiopetide antibiotic geninthiocin B (**20**) is due to the *Gen B* gene encoding a putative lantibiotic dehydratase in the biosynthetic gene cluster of the lichen-derived *Streptomyces* sp. YIM130001. As described in the literature, the production of associated genes includes precursor proteins, Yeao cyclodchydratase, lanthipeptide dehydratases, etc. (Figure 15A). The biosynthetic pathway of geninthiocin was proposed by Schneider et al. [28], and is exhibited in Figure 15B. The precursor peptide (*GenA*) unit, which possesses a 31 aa leader peptide (LP), is connected with a C-terminal 15 aa core peptide unit. The Yeao cyclodchydratase biosynthetic gene clusters *GenG1* and *GenG2* could catalyze the processing of azole rings formation. The proteins *GenB* and *GenC* show a high degree of similarity to lanthipeptide-like dehydratases and most likely catalyze the formation of the dehydroalanine (Dha) and dehydrobutyrine (Dhb) functional groups. The two Dha group residues from the serines Ser_1_ and Ser_13_ are then utilized by *GenD* for assembly of the central six-membered nitrogenous heterocycle. Finally, the cleavage of Ser_15_ to afford the C-terminus amide and the hydroxylation of Val_7_ is catalyzed by *GenI* and *GenH*, completing the biosynthesis of geninthiocin B.

**Figure 16:**
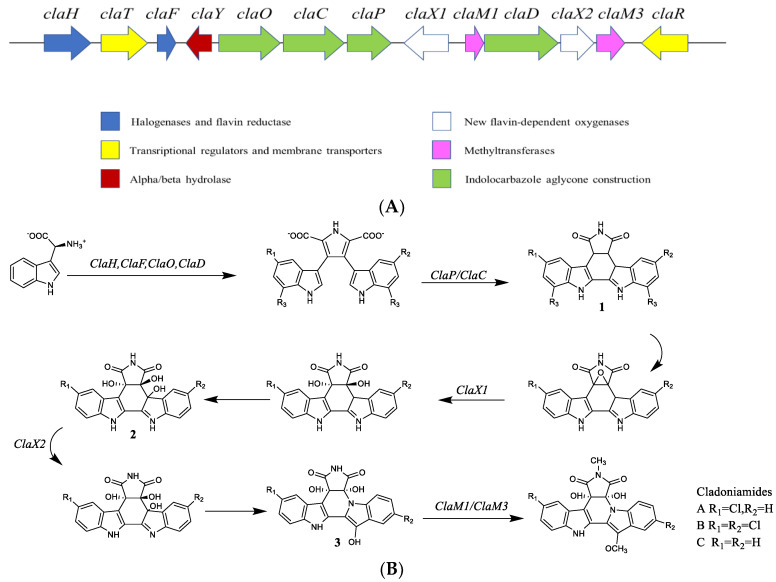
(**A**) The gene clusters of cladoniamides. (**B**) The biosynthetic pathway of cladoniamides A–C [74].

The genome of *Streptomyces uncialis* includes *halogenases and flavin reductase*, *indolocarbazole aglycone construction*, *new flavin-dependent oxygenases*, and so on (Figure 16A). The biosynthetic pathway of cladoniamides were proposed by Ryan et al. [74] and is exhibited in Figure 16B. It shows that *ClaH* and *ClaF* are highly related to the characterized L-tryptophan chlorinases, and that chlorine is installed on the L-tryptophan in the first step of the related rebeccamycin pathway. Due to the action of *ClaH* and a partner flavin reductase *ClaF*, L-tryptophan is chlorinated at the C-5′ position. Then, 5-chloro-L-tryptophan reacts with *ClaO*, generating an indole-3-pyruvate imine. *ClaD* dimerizes two of these molecules to generate a chromo pyrrolic acid molecule. *ClaY* catalyzes the hydrolysis of an amide bond in the N-methylsuccinimide ring, which is followed by oxidative decarboxylation. Three enzymes unique to the indenotryptoline biosynthetic pathway include two putative flavin-dependent oxygenases (*ClaX1* and *ClaX2*) and a putative α/β hydrolase (*ClaY*) shown in Figure 15A. The cladoniamide biosynthetic gene cluster is highly homologous to that of BE-54017. **1** (R_1_ = Cl, R_2_ = R_3_ = H) and a methylated derivative of **2** (R_1_ = R_2_ = H) separately accumulate in the BE-54017 heterologous expression system when the genes *abeX1* and *abeX2* are mutated, respectively. The route to generate downstream metabolites, indenotryptoline-containing molecules such as **3**, from substrate **2** via cleavage of the epoxide could be driven via ketone formation from one tertiary alcohol, causing sigma-bond rupture and epoxide hydrolysis, opening the indolocarbazole scaffold. This cleaved molecule could then close through attack on the ketone by the indolic nitrogen, restoring the tertiary alcohol and arriving at the indenotryptoline scaffold **3**. Each of these enzymes is thought to catalyze the transfer of a methyl group to a phenolic oxygen, consistent with the likely role of *ClaM3* in cladoniamide biosynthesis of installing a methyl group on the appended hydroxyl group to produce cladoniamides A–C.

## 6. Conclusions

The current review focused on lichen actinomycetota from four different perspectives. (1) Lichen-associated actinomycetes represent a promising but underutilized resource. A wide variety of novel actinomycetes have been isolated from lichens. (2) The potential of bioactive metabolites from lichen actinomycetes has been explored, and a total of 114 secondary metabolites from lichen-associated actinomycetes are summarized here. (3) Although the biological activities of many lichen actinomycetota have been investigated, their definite chemical components are still undetermined. Thus, the discovery of more novel bioactive compounds reveals new research prospects. (4) The biosynthetic pathways of some unique secondary metabolites isolated from lichen-derived actinomycetes are discussed.

It has become increasingly difficult to isolate new sources of actinomycetes from common environments such as the soil, sea, and plants. These resources no longer meet the increasingly urgent demand for new drug-leading compounds [6]. Therefore, researchers must explore potent microbial resources from unique environments [64]. Current research on lichen-associated actinomycetes has focused mainly on Asia, whereas lichens are globally distributed. This wide distribution range enables researchers to search for novel species. Lichen environments are understudied in terms of microbiology, but they should not be disregarded in the hunt for novel actinomycetota and their diversity of beneficial chemical compounds [71]. The novelty and variety of lichen actinomycetota are evident in this review. Furthermore, the study of biosynthetic pathways is a crucial process in the excavation of bioactive natural products [75]. Biosynthesis of other biologically active compounds is relatively less studied and requires more attention from researchers. Future work could attempt to reveal more silent biosynthetic gene clusters, so that to uncover more and more novel and interesting biologically active natural products from lichen-associated actinomycetota.

## Figures and Tables

**Table 1:** Novel actinomycetota taxa isolated from lichens between 2007 and 2022.

Family	Genus	Species	Lichen Habitats	Media	Refs.
*Aurantimonadaceae*	*Aureimonas*	*Aureimona leprariae*	Yunnan Province, south-west PR China	Humic acid–vitamin agar and ISP2	[8]
*Methylobacteriaceae*	*Methylobacterium*	*Methylobacterium planium*	Yunnan Province, south-west PR China	Humic acid–vitamin agar	[9]
*Microbacteriaceae*	*Glaciibacter*	*Glaciibacter flavus*	The south bank forest of the Baltic Sea, Germany	Humic acid–vitamin agar	[10]
	*Frondihabitans*	*Frondihabitans cladoniiphilus*	The natural spruce forest at Koralpe, in the Austrian Alps	Tryptone–yeast extract medium and ISP2	[11]
	*Leifsonia*	*Leifsonia lichenia*	The Botanical Garden of the University of Tokyo	Modified Detmer medium and nutrient agar	[12]
	*Naasia*	*Naasia lichenicola*	The south bank of the Baltic Sea, Germany	Humic acid–vitamin agar and YIM 38 medium	[13]
	*Schumannella*	*Schumannella luteola*	Tokyo, Japan	Modified Detmer medium and nutrient agar	[14]
	*Subtercola*	*Subtercola lobariae*	Jiaozi Snow Mountain, Yunnan Province, China	Potato dextrose agar and ISP2	[15]
*Micromonosporaceae*	*Actinoplanes*	*Actinoplanes lichenis*	Maha Sarakham Province, Thailand	Humic acid–vitamin agar with nalidixic acid and cycloheximide and ISP2	[16]
		*Actinoplanes lichenicola*	Maha Sarakham Province, Thailand	Humic acid–vitamin agar with nalidixic acid and cycloheximide and ISP2	[17]
		*Actinoplanes ovalisporus*	Maha Sarakham Province, Thailand	Humic acid–vitamin agar with nalidixic acid and cycloheximide and ISP2	[17]
*Nakamurellaceae*	*Nakamurella*	*Nakamurella albus*	Yunnan Province, south-west PR China	Humic acid–vitamin agar and ISP2	[18]
	*Nakamurella*	*Nakamurella leprariae*	Yunnan Province, south-west PR China	Humic acid–vitamin agar and YIM 38 medium	[19]
*Nocardioidaceae*	*Nocardioides*	*Nocardioides exalbidus*	Izu-Oshima Island, Japan	IAM-A1 agar medium and trypticase soy agar	[20]
*Promicromonosporaceae*	*Luteimicrobium*	*Luteimicrobium album*	Rishiri Island, Japan	Humic acid–vitamin agar with nalidixic acid and cycloheximide and nutrient agar	[21]
*Pseudonocardiaceae*	*Actinomycetospora*	*Actinomycetospora iriomotensis*	Iriomote Island, Japan.	Humic acid–vitamin agar with nalidixic acid and cycloheximide and nutrient agar	[22]
		*Actinomycetospora rishiriensis*	Rishiri Island, Hokkaido, Japan.	Humic acid–vitamin agar with nalidixic acid and cycloheximide	[23]
	*Pseudonocardia*	*Pseudonocardia*	Nahuel Huapi National Park, Patagonia	Artificial soil agar and Emerson’s yeast extract–starch agar and KEHE agar	[24]
*Rhodobacteraceae*	*Paracoccus*	*Paracoccus lichenicola*	Yunnan Province, south-west PR China	Humic acid–vitamin agar and YIM 38 medium	[25]
	*Rubellimicrobium*	*Rubellimicrobium rubrum*	The south bank forest of the Baltic Sea, Germany	Humic acid–vitamin agar and YIM 38 medium	[26]
*Streptomycetaceae*	*Streptomyces*	*Streptomyces lichenis*	Chiang Rai Province, Thailand	Arginine–vitamin agar and ISP2	[27]
		*Streptomyces*	The tropical rainforest in Xishuangbanna, Yunnan, China	YIM 212 medium	[28]
		*Streptomyces parmotrematis*	Doi Suthep-Pui National Park, Chiang Mai Province, Thailand.	Humic acid–vitamin agar and starch casein nitrate agar with cycloheximide and nalidixic acid	[29]
*Thermomonosporaceae*	*Actinomadura*	*Actinomadura violacea*	Pong Phra Bat Waterfall, Chiang Rai Province, Thailand	Humic acid–vitamin agar with nalidixic acid and cycloheximide	[30]
		*Actinomadura parmotrematis*	Chiang Rai Province, Thailand	Starch–casein nitrate agar with nalidixic acid and cycloheximide	[31]
